# Curricular and Administrative Transformation of the Nursing Program at Universidad de Antioquia. 75 Years of History

**DOI:** 10.17533/udea.iee.v43n3e17

**Published:** 2025-11-06

**Authors:** Elvigia María Posada Vera, Hugo Alberto Múnera Gaviria

**Affiliations:** 1 Nurse, Ph.D. Professor. Email: emaria.posada@udea.edu.co. https://orcid.org/0000-0003-3944-8788 Universidad de Antioquia Colombia emaria.posada@udea.edu.co; 2 Nurse, M.Sc. Professor. Email: hugo.munera@udea.edu.co https://orcid.org/0000-0001-8785-0304 Universidad de Antioquia Colombia hugo.munera@udea.edu.co; 3 Faculty of Nursing at Universidad de Antioquia; Medellín, Colombia. Universidad de Antioquia Faculty of Nursing Universidad de Antioquia Medellín Colombia

**Keywords:** nursing education, curriculum, history of nursing, students, nursing., educación en enfermería, currículo, historia de la enfermería, estudiantes de enfermería., Educação em Enfermagem, currículo, história da enfermagem, estudantes de enfermagem.

## Abstract

This essay describes the curricular and administrative transformation of the Nursing Program at Universidad de Antioquia since its founding in 1950, identifying progress and challenges up to current times in professional formation, institutional management, and care as disciplinary knowledge. The information has been organized by decades, which evidences historical processes, recognizes change patterns, and highlights relevant milestones more clearly, facilitating a critical analysis of academic evolution in long-term contexts. The institutional trajectory reveals an adaptive capacity regarding social and educational challenges. The most significant milestones include the transition from School to Faculty (1980), the high-quality accreditation from the National Ministry of Education (1999, 2006, 2013, and 2022), incorporation of high technology to teaching (2017), creation of a PhD degree in Nursing (2010), and strengthened work with international networks (as of 2022). These processes have consolidated a critical, humanistic, and socially relevant training in students and graduates. The recovery of this institutional memory strengthens the disciplinary identity and allows projecting a transformative nursing program into the future, committed to academic excellence, social justice, and educational innovation. The historical overview offers tools to address contemporary challenges in health and education, and reaffirms the Faculty’s role as a national benchmark in higher education in nursing.

## Resumen

Este ensayo describe la transformación curricular y administrativa del programa de Enfermería de la Universidad de Antioquia desde su fundación en 1950, identificando avances y desafíos hasta la actualidad en la formación profesional, la gestión institucional y el cuidado como saber disciplinar. La información se organizó por décadas, lo que permitió evidenciar procesos históricos, reconocer patrones de cambio y destacar hitos relevantes con mayor claridad, facilitando un análisis crítico de la evolución académica en contextos de largo plazo. La trayectoria institucional revela una capacidad adaptativa frente a los desafíos sociales y educativos. Entre los hitos más significativos se encuentran la transición de Escuela a Facultad (1980), la acreditación de alta calidad por el Ministerio de Educación Nacional (1999, 2006, 2013 y 2022), la incorporación de alta tecnología en la enseñanza (2017), la creación del Doctorado en Enfermería (2010) y el fortalecimiento de trabajo con redes internacionales (a partir de 2022). Estos procesos han consolidado una formación crítica, humanista y socialmente pertinente en estudiantes y egresados. La recuperación de esta memoria institucional fortalece la identidad disciplinar y permite proyectar a futuro, una enfermería transformadora, comprometida con la excelencia académica, la justicia social y la innovación educativa. El recorrido histórico ofrece herramientas para enfrentar los retos contemporáneos en salud y educación, y reafirma el papel de la Facultad como referente nacional en la formación superior en enfermería.

## Resumo

Este ensaio descreve a transformação curricular e administrativa do programa de Enfermagem da Universidade de Antioquia desde sua fundação em 1950, identificando avanços e desafios até o presente na formação profissional, na gestão institucional e no cuidado como domínio disciplinar. As informações foram organizadas por década, o que nos permitiu destacar processos históricos, reconhecer padrões de mudança e destacar marcos relevantes com maior clareza, facilitando uma análise crítica da evolução acadêmica em contextos de longo prazo. A trajetória institucional revela uma capacidade adaptativa diante dos desafios sociais e educacionais. Entre os marcos mais significativos estão a transição de Escola para Faculdade (1980), a acreditação de alta qualidade pelo Ministério da Educação Nacional (1999, 2006, 2013 e 2022), a incorporação de tecnologia avançada no ensino (2017), a criação do Doutorado em Enfermagem (2010) e o fortalecimento do trabalho com redes internacionais (a partir de 2022). Esses processos consolidaram uma formação crítica, humanística e socialmente relevante para estudantes e graduados. A recuperação dessa memória institucional fortalece a identidade disciplinar e nos permite projetar um futuro transformador para a enfermagem, comprometido com a excelência acadêmica, a justiça social e a inovação educacional. Este panorama histórico oferece ferramentas para enfrentar os desafios contemporâneos em saúde e educação e reafirma o papel da Faculdade como referência nacional no ensino superior de enfermagem.

## Introduction

Throughout its 75-year trajectory, the Nursing program at Universidad de Antioquia has been the scenario of profound curricular and administrative transformations that have consolidated ethical, pertinent, and scientifically rigorous professional formation. These processes have responded to the country’s social, political, and health challenges, strengthening institutional autonomy and raising the standards of academic quality. Curricular evolution has integrated research, critical, and humanistic skills, while administrative reforms have facilitated the accreditation of programs, internationalization, and participatory governance. As Parra warns,^1^ the nursing curriculum reflects tensions between disciplinary knowledge and social demands, so its transformation requires reflection, evidence, and historical commitment.

Within this context, this essay describes how said transformations, articulated to formative proposals aimed at a socially relevant graduation profile, have responded to academic, political, and professional demands, configuring specific care needs according to each period. For such, primary sources were consulted, such as publications by professors, specially the text by professor Cecilia Mabel Restrepo “History of the Faculty of Nursing at Universidad de Antioquia: an approach to the history of nursing in Antioquia. 1997”, who rigorously documents the history of the Nursing program since its creation in 1950 until the early 1990s; as well as meeting minutes, management reports from various deanships, focus group reports, and professor testimonies. The information was organized by decades to comply with the proposed objective and establish a historically coherent narrative. During the consultation process, the documents were kept with strict conservation measures, preventing their reproduction without due authorization.

### The 1950s: Founding of the School of Nursing at Universidad de Antioquia. Religious fundamentals, professionalization of care, and first steps toward autonomy.

The creation of the School of Nursing at Universidad de Antioquia in 1950 marked a milestone in the professionalization of care in Colombia. In response to the health needs of the time, the University’s Board of Directors approved, through Resolution 30 of 29 September, its establishment as a dependency of the Faculty of Medicine. The sociopolitical and cultural context of the time, influenced by medical positivism, Christian charity, and the empirical practice of health, shaped a training proposal that articulated technical knowledge with a religious vocation. The Dominican Sisters of the Presentation of Tours played a decisive role in consolidating this initiative, providing structure, discipline, and institutional vision.^2^

The School began its activities on 27 October 1950, at Colegio de la Presentación, under the direction of Sister Lucía de la Pasión, a nurse trained at Universidad Javeriana, with additional studies in Rochester and Baltimore. Her experience in academic organization was key for the initial curricular and administrative design. The government structure was conformed through a mixed Board of Directors, integrated by the dean of Medicine, two doctors and two nuns with nursing degrees recognized by the State. The institutional purpose reflected the vision of the time: to train religious personnel capable of collaborating with physicians in preserving health, with emphasis on moral and spiritual well-being.

In May 1951, the University requested from the National Ministry of Education the School’s official recognition, given that its creation had been internal. This recognition was formalized in 1961 through Agreement 31, which endorsed its study plan and legitimized the degrees issued. To resolve the ambiguity of the degrees issued during the first decade, it was decided to include the University’s main seal on the diplomas. In addition, the Academy of Medicine issued a Resolution backing the School’s scientific level of the School, urging institutions, like the Colombian Social Security Institute (ICSS, for the term in Spanish) and healthcare centers to offer job opportunities to its graduates. The official approval permitted access to publications from the National School of Nurses, facilitating the acquisition of scientific texts, scarce at the time. The School did not have its own library until November 1951, when a nursing section was authorized in the library of the Faculty of Medicine.

The training, which lasted three years, was based on biomedical theory, conventual discipline, and strict regulations. The students knew the rules and sanctions since their admission, and ensured compliance of such. The regime was so demanding that there were no vacations and shifts could extend up to 15 days without a break. Further, the religious community could designate or remove the nuns without consulting with the University. As of 1957, reforms began that paved the way for autonomy. On August 9 of that year, the School’s director chaired the Advisory Council for the first time, replacing the Dean of Medicine. In 1958, the reorganization of the Superior Council permitted including student representation, and the School voted on an outstanding third-year student. This generated a critical attitude among the students, highlighting the gap between university regulations and the conventual logic governing the School. Demands arose to adhere to the university calendar, modify the disciplinary regime, and reduce the academic load.

### The 1960s: Disciplinary rupture, curricular reform, and critical thinking: the decade that sowed the seeds of modern nursing.

During the 1960s, the School of Nursing at Universidad de Antioquia underwent a profound transformation, influenced by the national political context and by the university reforms promoted from abroad. In particular, the proposals promoted by US organizations to modernize Latin American universities introduced management models that restricted the democratic participation of students and professors.^3^ This trend was also reflected at Universidad de Antioquia, generating internal tensions that led to student and academic mobilizations. In the School of Nursing, these mobilizations led to a rupture with traditional disciplinary schemes. Students began to adopt more-critical stances regarding institutional regulations, influenced by emerging social movements, such as feminism and hippies, which permeated the university environment. Although the Advisory Council reaffirmed the existing standards through Agreement 17 of December 1961, students’ profiles changed significantly, moving away from the passive obedience that had characterized previous generations.

In 1963, the School began a curricular reorganization process in response to recommendations from international organisms, such as the World Health Organization (WHO), which pointed to deficiencies in the formation of nurses in areas like public health, research and teaching. This reform sought to align the curriculum with the country’s social needs, incorporating hygienic and health principles, and promoting a professional practice based on scientific knowledge and community care.

In 1965, following the guidelines by the Colombian Association of Nursing Faculties (ACOFAEN, for the term in Spanish), the complementary Bachelor’s Degree in Nursing program was created, with an additional year of training, through Agreement No. 1 of 15 January. This program sought to strengthen the graduate’s competencies in administration and teaching. For its implementation, an interdisciplinary School Council was comprised, made up of representatives from the faculties of Medicine and Education, as well as directors from the schools of Nursing, Public Health, and the Institute of General Studies. In addition, a research committee with its own regulations was established, recognizing this area’s importance to develop critical thinking and improve quality in nursing services.

Coexistence of basic and complementary programs implied significant administrative and academic adjustments. In 1966, the School adopted the university statutes, the institutional academic calendar, and the admissions exam as sole admission mechanism. The students began their first semester at the Institute of General Sciences, which marked a rupture with the model focused exclusively in the San Vicente de Paúl Hospital as practice scenario. This reorganization allowed the practices to be distributed by areas of care, in coherence with the new curricular focus. These changes reflect a philosophical transformation in nursing formation, aimed at preventive, community, and critical care, in tune with the social and academic demands of the period.

### The 1970s: Autonomy, secularization, and curricular reform: the decade that redefined institutional identity.

The 1970s marked a substantial advance in the consolidation of the academic and administrative autonomy of the School of Nursing at Universidad de Antioquia. In 1967, as part of the institutional modernization, participation by the director as president of the Advisory Council was made official. Nevertheless, it was in 1971 when full autonomy was granted to lead the Nursing program, which allowed her to initiate a progressive curricular reform, approved by the Board of Directors in 1970. This reform responded to the recommendations by the National Health Plan and by international organisms, and redefined the institutional philosophy, guiding the formation toward the development of critical awareness in professionals, emphasizing comprehensive care of individuals, families, and communities. The organic charter was modified, establishing new academic sections: basic sciences, medical-surgical, maternal-child, administration, psychiatry, public health, and continuing education. Internal committees were reorganized to address the specific needs of each area.

In the administrative setting, relevant progress was made. The director began attending Board of Directors meetings as a permanent guest, although without voting rights, due to her dependence on the Faculty of Medicine. In 1972, entry of faculty staff into the university hierarchy was authorized, and in 1974 the positions of deputy director and section heads were officially recognized, with their respective salary assignments. The national socio-political context also impacted the School’s internal dynamics. Increasing participation in university debates and openness to new currents of thought generated tensions among the different levels. In November 1974, the nuns submitted their final resignation from the academic and administrative leadership, marking the end of a period characterized by strong moral and religious influence. This event allowed greater freedom of institutional expression and gave way to a critical environment, with diverse political postures and administrative challenges faced by the first secular female dean.

Secularization as a path of uncertainty was evident with the departure of the nuns in 1974, an event that marked an institutional shift in the School of Nursing at Universidad de Antioquia and gave way to a stage of administrative and academic reorganization. In 1975, through Agreement 6 by the Superior Council, its separation from the Faculty of Medicine was officialized, remaining as a teaching unit under the Academic Direction. As of then, the School acquired autonomy to coordinate its programs, with support from a deputy director, a School Council, and an Advisory Council with student participation. Five departments were created: Fundamentals of Nursing, Medical-Surgical, Maternal-Infant, Administration of Nursing Services, and Continuing Education. In 1978, Agreement 13 authorized its internal subdivision by sections, based on the academic and administrative load. For example, The Medical-Surgical Department was organized into internal medicine, surgery, psychiatry, and mental health; the Maternal-Child Department into obstetrics and pediatrics; and the Administration Department into hospitalization and outpatient care.

Department chiefs were assigned by the Board of Directors, based on the director’s proposals, and each established its own regulatory council. However, the School faced an abrupt expansion in the number of students, going from 467 in 1974 to 914 in 1975, without increasing proportionally the physical resources, professors, or practice scenarios. In the absence of defined criteria for hiring professors, the Academic Council expedited Resolution 09 of 1976, establishing minimum requirements, such as professional degree, specific experience, and skills for collaborative work. This measure sought to guarantee academic quality amid a growing demand. Pressure on the practice scenarios led the health institutions to limit the number of students per professor. The lack of formal agreements between the university and healthcare services generated operational tensions. Only until Decree 1210 of 1978 were teaching and healthcare activities regulated, allowing agreements between universities and health entities. 

In 1979, The School formalized the coordination of practice fields through Resolution 16 of the Academic Council. As a pedagogical response, the skills laboratory was created, which was a simulation space that complemented clinical practice. At the same time, regulatory changes in health and education required a profound curricular reform. Incorporation of concepts, like health/illness, community, culture, and human development, along with the institutionalization of mandatory social service (Decree 2184 of 1976 and Resolution 2050 of 1977), justified the inclusion of rural practice in the final year of the program. Nursing formation began to be guided toward the social and collective, with an intersectoral and interdisciplinary approach. This transformation encouraged a critical reflection on care focused exclusively on the clinical aspect, promoting a broader vision of the health-society relationship. Qualitative research gained relevance, strengthening the epistemological debate and becoming consolidated during the following decade. The curricular reform was structured over a new philosophical framework that integrated the clinical, social, and community aspects. The study plan was organized by levels of complexity, considering individuals in their social context and promoting prevention, recovery, and rehabilitation. For its implementation, teacher training was strengthened by the Department of Continuing Education, which in 1978 focused its efforts on preparing the faculty staff under the new conceptual guidelines.

### The 1980s: From School to Faculty: academic autonomy, curricular reform, and research expansion.

The 1980s marked an inflexion point in the academic consolidation of the School of Nursing at Universidad de Antioquia. In 1980, the Colombian Institute for the Promotion of Higher Education (ICFES, for the term in Spanish) endorsed a new four-year curricular plan, structured into eight semesters, which replaced the complementary bachelor’s program, closed in 1979. This plan was approved by the University through Resolution 026 of 11 December 1980 and ratified by the ICFES with Agreement 046 of 24 February 1981. In parallel, the Superior Council recognized the School as a teaching unit with academic autonomy, transforming it into Faculty through Agreement 10 of 1980. This decision involved an organizational restructuring formalized in Agreement 11 of 9 May 1981, which established a deanship, a vice-deanship and four departments: Comprehensive Adult Health Care (AISA, for the term in Spanish), Maternal and Child Care, Health Administration, and Research and Education. Each was subdivided into specific sections, responding to the training and operational needs of the Faculty.

Implementing the new plan required defining a professional profile for the students, as condition established by the ICFES to certify the program. Moreover, the teaching-caring relationship was strengthened through the creation of the Hospital-University Coordinating Committee, which promoted articulation between health institutions and the Faculty. The professors actively participated in SENA committees and hospitals in Medellín, which facilitated the formalization of practice scenarios through institutional contracts. In the midst of a turbulent social context, professors began reflective processes on their professional work, which led to significant research. These efforts drove the creation of an academic journal and a research center (1987), which later obtained national and international recognition. In addition, extension services in advisory, consulting, and training were consolidated, and the foundations were laid for the first graduate programs.

The institutional crisis of 1985, which led to the temporary closure of the University by decision of the Superior Council, motivated an overall restructuring. In 1986, the Academic Council issued Agreement 2, establishing guidelines to reform the curricular plans. This initiative was articulated with the self-assessment project promoted by the Colombian Association of Faculties of Medicine (ASCOFAME, for the term in Spanish), the Colombian Association of Nursing Faculties (ACOFAEN, for the term in Spanish), and the Association of Faculties of Dentistry (ACFO, for the term in Spanish), which called on health faculties to review their programs in light of new social demands. The Faculty of Nursing actively participated in the process, developing internal debates about professional functions, thinking, and practice. As a result, the objectives of the undergraduate program were redefined, and the study plan was reorganized into three components: core subjects, professional subjects, and complementary subjects. This proposal was approved by the Faculty Council in early 1989. Finally, the Academic Council issued Resolution 1001 of 1989, delegating in the Faculty councils the partial modification of study plans, approval of academic calendars, and formation of curricular committees, subject to approval by the Academic Directorate. This measure streamlined academic-administrative processes and strengthened the faculties’ curricular autonomy.

### The 1990s: Regulatory reforms, pedagogic innovation, and institutional consolidation: the decade that redefined nursing.

During the 1990s, Colombia underwent a regulatory reconfiguration in health and education sectors that directly impacted the nursing formation. Legislation 30 of 1992 introduced a pedagogical approach focused on students as active, autonomous, and critical subjects, promoting more comprehensive and participative educational models.^4^ This transformation required institutions to overcome rote learning and adopt perspectives that recognized the complexity of human beings in their formative process. Within this context, the Faculty of Nursing at Universidad de Antioquia undertook a thorough review of its academic program. Since 1991, the Prospective Analysis and Normative Model Group proposed a curricular update that would respond to social, normative and disciplinary changes, integrating diverse knowledge and redefining the institutional educational philosophy. This proposal was consolidated in the Institutional Educational Project (PEI, for the term in Spanish), which guided formation toward a graduate profile with scientific, technical and social skills, capable of practicing nursing in a critical and reflective manner.

The new curriculum, lasting eight semesters, was structured into three lines: Basic Foundation, Professional Foundation, and Advanced In-depth Foundation. The latter allowed students to specialize in a management area, strengthening their skills as caregivers, researchers, managers, and educators. The conceptual framework was organized around categories, like the human being, care, environment, and the human vital process, with nursing care as articulating axis of the formative process.

At the same time, a significant administrative transformation was carried out. Superior Agreement 005 of 1994 redefined the Faculty’s organizational structure; the Dean’s Office was established as the head, followed by the Vice-Dean’s Office, the Curriculum Committee, the Planning Committee, and the Faculty Council. The departments of Basic Professional Training, Professional Training, Extension and Graduate Studies, as well as the Research Center, emerged from the latter. Professors from the first departments supported the extension, research, and graduate processes according to projects underway. Key bodies were strengthened, such as the Tutoring Committee (Resolution 042 of 1990), the Curriculum Committee (Agreements 007 of 1992 and 010 of 1994), the Concertation Committee (Resolution 114 of 1993), and the Professor Performance Evaluation Committee (Resolution 102 of 1992), all under the direction of the Faculty Council. These instances guaranteed participation from professors, students, and graduates in academic decision-making, promoting collegial management. As result of this progress, on 17 March 1999 the Faculty obtained for the first time the high-quality accreditation granted by the National Ministry of Education, recognition that has been renovated in 2006, 2013, and 2022.

A significant achievement was the creation of the first graduate programs: three clinical specializations and an interdisciplinary master’s in Collective Health. These programs responded to the conceptual strengthening of nursing as a discipline and profession, and to the advancement of research with greater theoretical and methodological maturity. The Faculty was positioned nationally and internationally, increasing its participation in academic events and administrative structures of entities such as ACOFAEN and the Latin American Association of Nursing Faculties (ALADEFE, for the term in Spanish), from where it influenced strategic decisions for the development of nursing in Colombia and Latin America. Another important achievement was the creation and graduation in 1999 of the first class of the program “University Training of Nursing Aides”, a program that responded to the call and the need for professional formation of this human resource. In 1998, the Faculty’s publishing project was approved, aimed at publishing scientific texts and articles in the discipline; today, it is the Journal *Investigación y Educación en Enfermería*. This initiative responded to the growing intellectual productivity of faculty, students, and graduates, and called for a clearer administrative structure, with greater dedication to teaching and research support.

During this decade, interinstitutional projects were also consolidated, strengthening the teaching-care relationship and the Faculty’s academic visibility. These include the Metropolitan Area Interinstitutional Nursing Committee (COINDEN, for the term in Spanish), with participation by nursing directors from healthcare institutions; the multidisciplinary teaching-care research program in the northeast region; and the UNI project, which articulated the university, community, and social system of the municipality of Rionegro. These initiatives were led by or had leading representation by the Faculty. In addition, ties with institutions were strengthened through training, consulting, internships, and the Nursing Academic Meetings (RAE, for the term in Spanish), consolidating a more structured Faculty in administrative, academic, and scientific terms. In sum, the 1990s represented a period of profound curricular and administrative transformation for the Faculty of Nursing at Universidad de Antioquia. Articulation among national regulations, disciplinary advances, and institutional vision allowed to consolidate a comprehensive educational model, strengthen the organizational structure, and project the Faculty as an academic benchmark in the country and the region.

### Decade from 2000-2010: a decade of disciplinary expansion, regional articulation, and strategic internationalization.

During the first decade of the 21^st^ century, the Faculty of Nursing at Universidad de Antioquia consolidated a curricular transformation that strengthened the professional profile in individual and collective care, as well as in socio-political research. Ties with the healthcare sector were strengthened through the RAE, the COINDEN of Antioquia, and participation of graduates in academic groups.

Growth of research groups, four of them classified by COLCIENCIAS, drove the disciplinary development. Continuing education and postgraduate programs were expanded, including specializations, master’s degrees, and the opening of the first PhD in Nursing cohort in 2010.^5^ The undergraduate high-quality reaccreditation for seven years and the qualified registration for regional programs evidence the institutional quality.

The professionalization of nursing aides and technicians was strengthened in regions, like Bajo Cauca and Urabá, and the Primary Health Care (APS, for the term in Spanish) project was implemented in 2006, 2007, and 2010 in agreement with the department of Antioquia, which permitted its presence in all the regions of the department. The teaching-service relationship was formalized through the Self-Regulation Model approved in 2004, and the Network of Nursing Practice Coordinators (REDECOPE, for the term in Spanish) was created. This strategy was created and led by the Faculty of Nursing at Universidad de Antioquia to articulate nursing training institutions around the teaching-service relationship.

Internationalization was reflected in student mobility with Mexico and Canada, and in the creation of the Interinstitutional Cooperation Committee. Finally, constant administrative adaptation allowed for the articulation of substantive functions and strategic planning under Superior Agreement 255 of 2003.

### Decade of 2011-2020: Academic consolidation and territorial expansion: recent milestones in the Faculty of Nursing

During this decade, the Faculty of Nursing at Universidad de Antioquia materialized significant progress in its curricular and administrative transformation. Highlights include the opening of the program in Eastern Antioquia, the first cohort of the Master’s in Collective Health in Urabá, and the expansion of continuing education in three regions of the department. Participation in the formulation of public policies on caregivers positioned the Faculty as a relevant actor in the city.

The first PhDs in nursing graduated, the specialization in cancer patient care was consolidated, and the skills laboratory was enhanced with high-tech simulators. Internationalization was evidenced by the increase in internships and student mobility. The pandemic boosted strategies, such as UdeA Nursing takes care of you, reaffirming the faculty’s social commitment to its academic community and society in general.

The curricular reform proposed in 2019 extended the undergraduate program to five years, with greater emphasis on areas, like family and community health, mental health, care in old age and aging, political training, management and research, as well as learning a second language. The pedagogical model adopted is based on the integration of knowledge, innovation, and flexibility, giving students an active role in their training process. These changes generated administrative impact with the creation of committees, relocation of staff, and more rigorous academic demands.

### Period of the years 2021-2025: Educating for caring in a changing world regarding the challenges of the digital era.

During the five-year period 2021-2025, the Faculty of Nursing at Universidad de Antioquia has consolidated important academic and administrative progress with a strategic vision. The implementation of the new ten-semester study plan, a proposal endorsed by the National Ministry of Education, began through Resolution 24208 of 23 December 2022, year in which the Faculty also again received the high-quality accreditation for eight years. During this implementation, the curriculum was strengthened, the use of technological tools as teaching strategies was strengthened, and the proposal to expand the educational offer in the regions of the Department of Antioquia was reactivated. The Specialization in Maternal Perinatal Nursing was created, and internationalization and interculturality processes were promoted in teaching, research, and extension. Project management permits improving the attainment of resources, while financial analysis became a key axis of discussion to face one of the biggest economic crises of the university. The Faculty’s virtual campus was developed, and the institutional journal was positioned as the most prominent in Latin America. Likewise, the administrative structure was adapted to respond to post-pandemic challenges and new ways of teaching and learning, in an environment marked by technology and artificial intelligence and with a generation of teachers, students, and graduates committed to knowledge, science, and new ways of caring. This progress has permitted the Faculty to actively join international nursing and health networks, as well as signing agreements with universities in other countries and regions. Said alliances have facilitated academic exchange, advisory services, consultancies and other international cooperation activities that enrich institutional training and projection. 

In synthesis, the first quarter century has represented for the Nursing program at Universidad de Antioquia a stage of consolidation and permanent renovation. Amid a confusing and challenging environment, the institution has responded with strategic vision, academic rigor, and social commitment, reaffirming its leadership in the formation of nursing professionals with critical, ethical, and transforming capacity.

### Final reflections

The trajectory of the Nursing program at Universidad de Antioquia, throughout its 75 years, evidences sustained commitment with academic excellence, social pertinence, institutional transformation. This process has been possible due to the participation of all its members, who have responded with openness, continuous training, and willingness to change in the face of the challenges of each era. Curricular advances have required adjustments in content and methodologies, as well as a flexible attitude by professors and students. This flexibility, understood as organizational and pedagogical principle, must be extended to administrative processes, communication channels, and to the regulatory interpretation, generating conditions that favor academic development and student wellbeing. In contemporary contexts, where students assume multiple social roles, this institutional adaptability becomes indispensable.

The Faculty’s evolution has been marked by strategic decisions that have permitted its transition from School to autonomous academic unit, with capacity to respond to social, university, and disciplinary demands. In this sense, the nursing formation has been oriented toward autonomy, quality, and social justice. In the current context, characterized by globalization, academic mobility, recognition of degrees, multi-center research, and curriculum internationalization, the Faculty faces new challenges. Inter-institutional cooperation, strengthening of applied research, knowledge management, and the ethical incorporation of emerging technologies - including artificial intelligence - are elements that must be critically integrated into professional training.^6^

The Faculty’s recent history also reveals a commitment to pedagogical innovation, expansion of educational offerings in the regions, strengthening of graduate programs, and consolidation of international networks. These processes have been accompanied by administrative restructuring that seeks greater functionality, coherence with the University’s General Statute, and articulation with the current substantive functions of teaching, research, and extension. 

The historical reconstruction of the processes occurring as of the 21^st^ century faces an important limitation: scarce academic bibliography available on the recent evolution of the Faculty. A good part of this narrative has been constructed from the direct experience of the authors, testimonies from professors, and unpublished institutional documents. This situation evidences the need to advance in documentary systematization and in academic production about the contemporary institutional history.

In conclusion, since its founding to the present, the Nursing program at Universidad de Antioquia has managed to consolidate itself as an academic benchmark in Colombia and Latin America, given its great capacity for curricular and administrative transformation. Its history not only narrates an organizational evolution, but also the commitment made to care as a critical, humanistic, and socially relevant practice. Within a challenging global environment, recovering this institutional memory is key to project a Nursing program committed with equity, innovation, and academic excellence. The future requires continuing to strengthen research, internationalization, and organizational flexibility, without losing sight of the ethical and emancipatory horizon that has guided its development.
